# Aptamer based surface plasma resonance assay for direct detection of neuron specific enolase and progastrin-releasing peptide (31-98)†

**DOI:** 10.1039/d1ra05041a

**Published:** 2021-09-29

**Authors:** Linlin Sun, Kemin Shen, Jianbin Zhang, Wenjuan Wan, Wenjun Cao, Zhijun Wang, Chongzheng Guo

**Affiliations:** Department of Preventive Medicine, Changzhi Medical College Changzhi Shanxi 046000 China sunlinlin@czmc.edu.cn +86-355-3151068; Department of Chemistry, Changzhi University Changzhi Shanxi 046011 China

## Abstract

Neuron specific enolase (NSE) and progastrin-releasing peptide (31-98) (ProGRP31-98) are considered as reliable biomarkers of small cell lung cancer (SCLC). Sensitive determinations of NSE and ProGRP31-98 show great significance in disease surveillance, clinical diagnosis, efficacy evaluation and prognostic judgment. However, the conventional detection methods have the disadvantages of poor stability, tedious operation, and being very time consuming. Herein, we developed an aptamer-based surface plasmon resonance (SPR) assay in a direct format for NSE and ProGRP31-98 detection. The aptamer was loaded on a sensor chip and used as an affinity ligand. With sample injection, SPR signals increased due to the association of the target to the aptamer coated chip. Further dissociation and regeneration allowed this aptamer sensor chip to be used for the next sample analysis. We achieved sensitive detection of NSE and ProGRP31-98 by measuring the affinity binding-induced SPR responses. The detection limits for NSE and ProGRP31-98 were 3.9 nM and 15.6 nM, respectively. The aptamer sensor chip is stable and reusable, and has potential for diluted human serum analysis. This assay presents strengths in simplicity, rapidity, low material consumption, real time analysis and ease of implementing high throughput and automatic detection. It is promising for application in clinical disease-related biomarkers analysis.

## Abstract 2

Direct detection of neuron specific enolase and progastrin-releasing peptide (31-98) were achieved by measuring the target-induced SPR response of an aptamer-coated chip.

## Graphical abstract

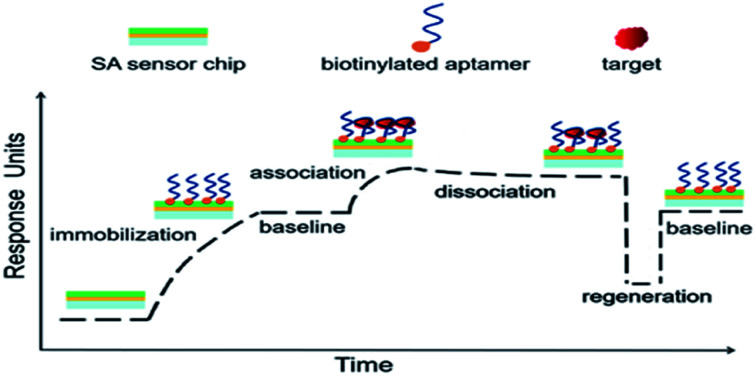

## Introduction

Lung cancer has been considered one of the most serious diseases threatening human life and health due to high mortality rate and a low overall 5 year survival rate.^1,2^ It is reported that the accurate determinations of disease-related biomarkers contribute to early diagnosis and increase survival.^3,4^ Neuron specific enolase (NSE) and progastrin-releasing peptide (31-98) (ProGRP31-98) have been reported to be good and reliable diagnostic biomarkers for small cell lung cancer (SCLC).^5^ NSE is a glycolytic neurospecific isoenzyme of enolase, found in nerve tissues and neuroendocrine tissues. Excessive NSE expression occurs in tumors associated with the origin of neuroendocrine tissues, especially SCLC, leading to a significant increase of serum NSE concentration.^6^ ProGRP31-98 is a hormone which is frequently produced by SCLC cells.^7^ The sensitive and accurate determinations of NSE and ProGRP31-98 show great significance and high potential in disease surveillance, early diagnosis and prognostic judgment of lung cancer. Until now, the most common detection methods for NSE and ProGRP31-98 analyses are immunoassays, including radioimmunoassay, enzyme-linked immunoassay (ELISA), chemiluminescence immunoassay, electrochemical immunoassay, and *etc.*^8–10^ These assays achieve the sensitive detection of NSE and ProGRP31-98, while the application of antibody presents disadvantages of tedious preparation, complex operation, poor reproducibility and limited storage condition. Other detection methods based on large analytical instruments, like liquid chromatography tandem mass spectrometer (LC-MS), are time-consuming and cumbersome to operate.^11^ Therefore, it is essential to develop new assays with high sensitivity, simple operation, rapid detection, and high repeatability and stability for NSE and ProGRP31-98 detection.

Aptamers, regarded as chemical antibodies, are single-stranded DNAs or RNAs which can bind to targets with high binding affinity and specificity.^12,13^ Compared to antibodies, aptamers possess some unique merits, including ease to chemical synthesized and modified *in vitro*, good thermal stability, high reproducibility and low or even no immunogenicity.^14^ These features allow aptamers to be widely used as affinity ligands in sensing analysis.^15^ Aptamers against various targets, like small molecules,^16^ heavy metals,^17^ proteins,^18^ virus,^19^ cells^20^ and *etc.*, have been reported and used for constructing biosensors and bioassays. Zheng *et al.*^21^ described the first successful selection of several DNA aptamers with high affinity against NSE, in which the aptamer of Apt-5 showed the highest binding affinity. By using Apt-5, they developed a sandwiched chemiluminescence aptasensor for NSE detection in serum samples. Aptamers showed strong affinity to ProGRP31-98 were also reported.^22^ By using a label-free electro-chemiluminescence measurement, the aptamers could detect ProGRP31-98 as low as 17 nM.^22^ The successful selecting of aptamers make it possible to construct aptamer-based assays for NSE and ProGRP31-98, while the assays are still limited. Chen *et al.*^23^ constructed a sandwich-structured electrochemical sensor for NSE detection by using a pair of aptamers.

Besides the above electrochemical assays, optical sensing technologies, like surface plasma resonance (SPR), biolayer interferometry (BLI), grating coupled interferometry, resonant waveguide gratings, and optical waveguide lightmode spectroscopy (OWLS), are gradually showing great applications in the study of molecule interactions and bioanalysis.^24–26^ Among these, surface plasma resonance (SPR) is known as a powerful golden standard for studying molecular interactions. SPR technique monitors the refractive index change occurred on the sensor chip, which is associated with the affinity binding of analyte to the immobilized ligand.^27,28^ Due to the features of label-free, real time and flexibility in design, SPR has been employed in the field of life sciences,^29^ drug discovery,^30^ environmental monitoring,^31^ food safety^32^ and *etc.* For bioanalysis, SPR allows not only the reliable and sensitive detection of targets, but also the exploration of kinetic studies. Different SPR biosensors have been constructed by adopting direct, sandwiched and competitive strategies. Compared to sandwiched and competitive methods, direct assays are much more desirable for the advantages of simplicity and rapidity.^33–35^ To our knowledge, SPR analysis for NSE and ProGRP31-98 detection has not been reported.

In this assay, we immobilized the aptamer on SPR sensor chip and regarded aptamer as affinity ligand. The integration of aptamer with SPR technique is expected to provide a novel and simple method for disease-related biomarkers detection. With sample injection, the target bound to aptamer and caused the increase of SPR signal as the flow of sample. Then the signal decreased with the dissociation of target. After regeneration, the aptamer coated chip was then used for next sample analysis, and the SPR responses were monitored in real time. We achieved successful detection of NSE and ProGRP31-98 by measuring the SPR signals induced by targets. Under optimized conditions, the detection limit of NSE and ProGRP31-98 were 3.9 nM and 15.6 nM, respectively. NSE and ProGRP31-98 detection in complex sample matrix were evaluated. Benefited from the advantages of aptamer, this assay shows good repeatability and stability without losing performance. This assay possesses good generality for other disease-related biomarkers detection.

## Experimental section

### Materials and reagents

The biotinylated aptamers and a random sequence of DNA were synthesized and purified by Sangon Biotech. Each had one biotin label at the 5′ terminal of sequence with a triethylene glyco (TEG) spacer. The anti-NSE aptamer and anti-ProGRP31-98 aptamer were noted as NSE-Apt5-5BioTEG and ProGRP-48-5BioTEG, respectively. The random sequence of DNA was used as DNA control. The sequences were shown as followings.

NSE-Apt5-5BioTEG: 5′-biotin-TEG-TCACACACGGACCTCTCCTACATTAATTGCGCATTTCGTT-3′.

ProGRP-48-5BioTEG: 5′-biotin-TEG-CATGCGGAGTAGATTCGAGCCCAGATAGTCCCTGGTTATTTCCTTAGG-3′.

DNA control: 5′-biotin-TEG-TCAGGTGCAGTTCTCGACTCGGTCTTGATGTGGGT-3′.

Recombinant neuron specific enolase (NSE) protein, progastrin-releasing peptide (31-98) (ProGRP31-98) and alpha-fetoprotein (AFP) antigen were ordered from Shanghai Linc-Bio Science Co. Ltd. Recombinant human carcinoembryonic antigen (CEA) was bought from Sangon Biotech. Recombinant human CA125 and recombinant human mucin-1 (MUC1) were ordered from Solarbio Life Sciences. Sodium dodecyl sulphate (SDS, 0.5%) was purchased from GE Healthcare. Working standard NSE and ProGRP31-98 solutions were freshly prepared from the concentrated stock solution by sequential dilutions in running buffer. Normal human serum was obtained from Shanxi Cell Biotech Co. Ltd. All solutions were prepared using ultrapure water obtained through a Purelab Ultra Elga Labwater system. All other reagents were of analytical grade.

### Preparation of aptamer coated sensor chip

For the performing of SPR measurements, SPR instrument (BIAcoreX100, brought from GE Healthcare) and streptavidin (SA) sensor chip (GE Healthcare) were introduced. 1 × PBS, pH 7.5, 5 mM MgCl_2_ and 0.1% Tween 20 was employed as running buffer for NSE detection. Typically, all solutions were filtered with 0.22 μm membrane before used. The aptamer coated sensor chip was prepared according to the following steps. Firstly, the mixture of 50 mM NaOH and 1 M NaCl was injected for 60 s at a flow rate of 10 μL min^−1^ to wash the SA sensor chip. After washing for three times, the running buffer was passed over the surface for 300 s at a flow rate of 10 μL min^−1^. Then, 20 nM biotinylated aptamer (NSE-Apt5-5BioTEG), diluted by running buffer, was injected for 300 s at a flow rate of 5 μL min^−1^. The loaded of aptamer on SA sensor chip was achieved through the strong binding of SA and biotin (*K*_d_ ∼ 10^−14^ M). It should be noted that aptamer was only captured on flow cell 2, and the flow cell 1 was used as reference cell.

### Surface plasma resonance (SPR) assay

Prior to analysis, the whole flow path of aptamer coated chip was primed by running buffer for three times. Different concentrations of NSE were diluted by running buffer and the solutions were injected for 180 s at a flow rate of 30 μL min^−1^. The binding of NSE to aptamer coated chip was monitored in real time. After each injection, flow of running buffer was continued for 600 s to allow the dissociation of target. The regeneration was then performed by injecting 0.5% SDS for 30 s. Report points were read at 4 s in relation to the end of injection in each cycle and each sample was tested for three times. The data were processed by Biacore X100 Evaluation Software.

## Results and discussion

### Assessment of this aptamer based SPR sensor

**Scheme 1:**
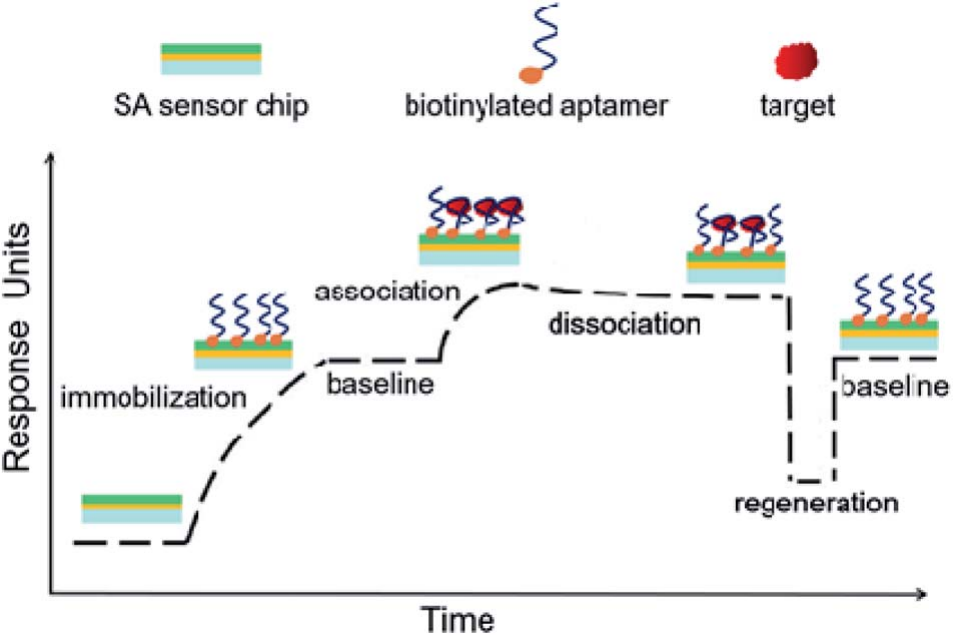
A schematic illustration for the principle and process of aptamer-based SPR assay in a direct format. Aptamer is immobilized on streptavidin (SA) sensor chip through the strong binding of biotin to SA. With the injection of sample, SPR response increases with the association of aptamer with target. Then a flow of running buffer induces the dissociation of aptamer–target complex. A further regeneration step allows SPR response back to baseline, and the aptamer coated chip can be used for next sample analysis.

Scheme 1 presents the principle and process of this proposed aptamer based SPR sensor in a direct format. Firstly, the aptamer coated SPR chip is prepared by utilizing the strong affinity binding of biotin to SA sensor chip. The immobilization of aptamer can be monitored through the increase of SPR signal. After a period of baseline, the sample solution is injected, and SPR response increases due to the affinity binding of target to the aptamer coated chip. Then the passing over of running buffer leads to the dissociation of target, along with the slightly decreases of SPR response. Finally, a regeneration step is conducted to regenerate the affinity surface, and the SPR signal is expected to return to the baseline for next sample analysis. According to this, the detection of target can be achieved by measuring the SPR signal induced by target binding.

**Fig. 1:**
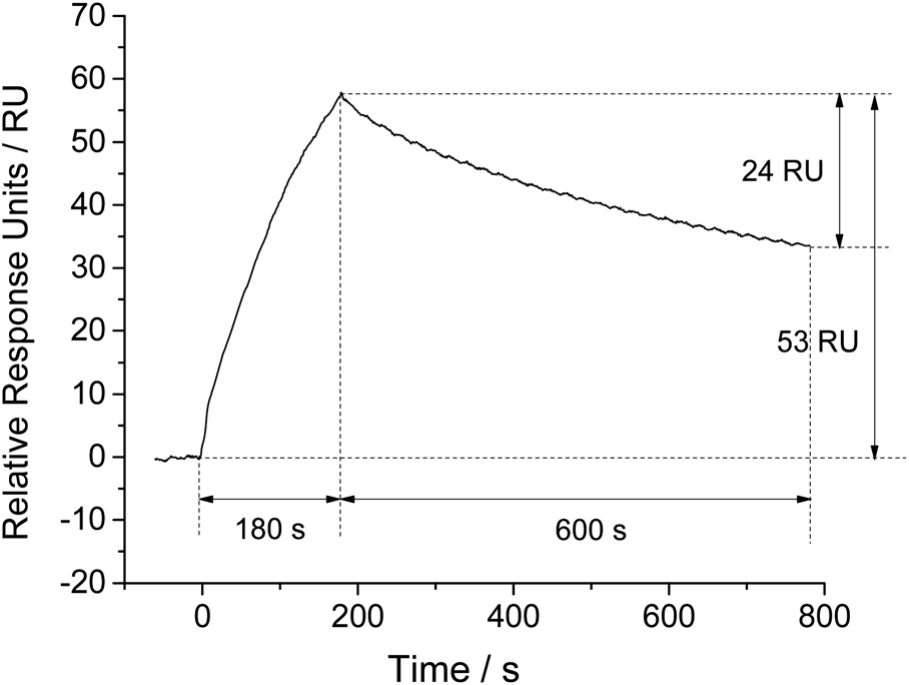
Association and dissociation kinetics of NSE (500 nM) to aptamer coated SPR chip, expressed as variation of response with time.

We took NSE as an example for feasibility assessment. The final amount of NSE-Apt5-5BioTEG bound was 170 RU (Fig. S1 in ESI†). After the preparation of aptamer coated chip, NSE samples were injected. The affinity binding of NSE to aptamer led to significant increase of SPR response, as the refractive index of aptamer coated chip was altered. As shown in Fig. 1, 500 nM NSE was injected for 180 s at a flow rate of 30 μL min^−1^. The SPR signal increased with the injection of NSE, indicating the affinity binding of NSE to aptamer coated chip. The final bound amount of 500 nM NSE was about 53 RU. The relative response units were calculated by subtracting the response units of reference cell (flow cell 1) from that of sample cell (flow cell 2). After a period of dissociation (600 s), the SPR signal decreased about 24 RU, which presented a relatively strong binding of NSE to aptamer. A further step of regeneration was needed to regenerate the chip surface. These results show it is feasible for NSE assay by using this aptamer-based SPR assay in a direct format.

### Regeneration test

**Fig. 2:**
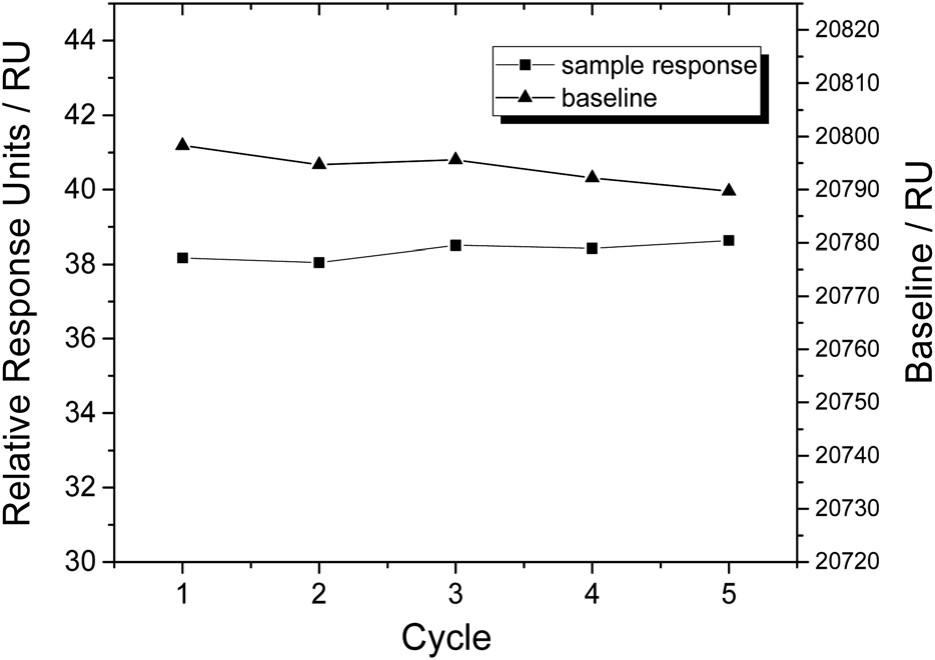
Regeneration test of aptamer coated sensor chip by SDS (0.5%), expressed as SPR signals of NSE samples (300 nM) and baselines in five cycles.

In order to test the regeneration effect of SDS (0.5%), we injected NSE (300 nM) for 180 s at a flow rate of 30 μL min^−1^. After a dissociation of 600 s, the chip was regenerated for 30 s by injecting SDS (0.5%) at a flow rate of 30 μL min^−1^. The association, dissociation and regeneration steps were repeated for five cycles under the same condition, and the SPR signals of baselines and samples were recorded (shown in Fig. 2). Within five cycles, SPR responses of aptamer coated chip to NSE samples were almost constant, and the baseline signals decreased slightly. The results indicate that our prepared aptamer coated chip can be regenerated well by SDS (0.5%) without losing binding affinity. 0.5% SDS was used for regeneration during the following experiments.

### MgCl_2_ effect

**Fig. 3:**
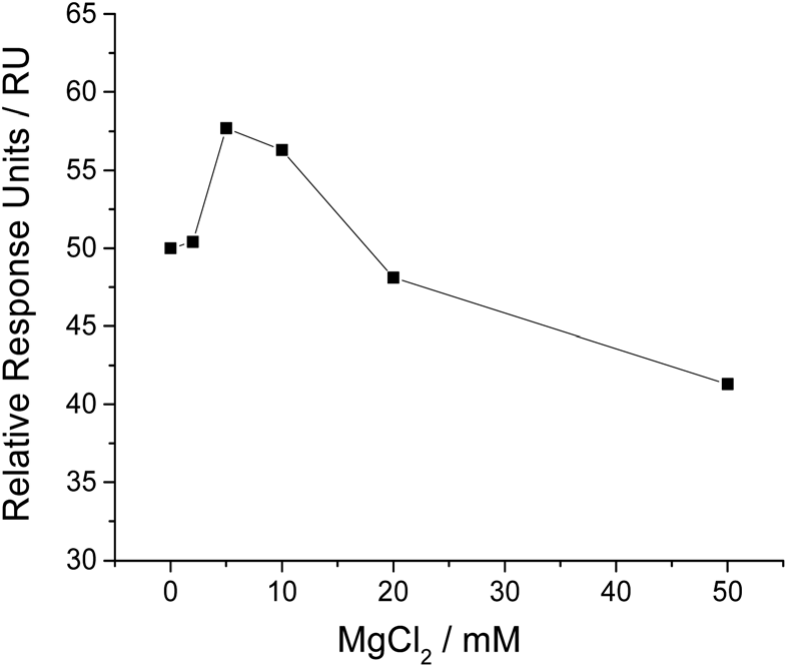
Influence of MgCl_2_ in running buffer on NSE detection, expressed as the relative SPR response units induced by NSE (500 nM) in running buffers containing 1 × PBS, pH 7.5, 0.1% Tween 20 and different concentrations of MgCl_2_.

It is reported that magnesium ion (Mg^2+^) plays a vital role in the binding of aptamer to target, as Mg^2+^ may be required for aptamer to form suitable conformation and maintain good binding affinity to target.^36^ In order to achieve the sensitive analysis of NSE, we investigated the influence of MgCl_2_ in running buffer on SPR responses. The results (shown in Fig. 3) suggest that a proper concentration of MgCl_2_ is necessary for sensitive analysis of NSE. We added 5 mM MgCl_2_ in running buffer, as it gave the highest relative SPR signal.

### NSE detection

**Fig. 4:**
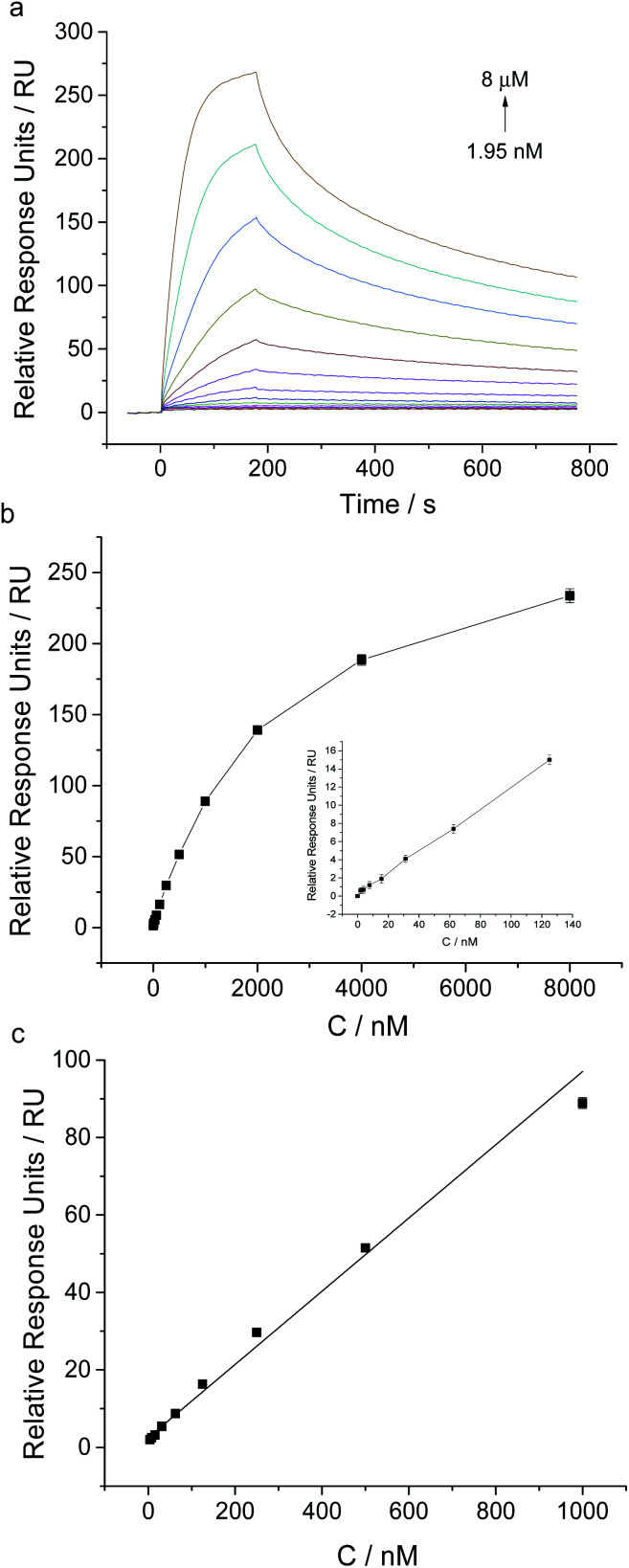
SPR analysis of different concentrations of NSE using the aptamer coated chip. (a) Sensorgrams of the injection of NSE at different concentrations ranging from 1.95 nM to 8 μM. (b) SPR response plot of the aptamer coated chip to different concentrations of NSE (inset: low concentrations ranging from 0 to 125 nM). (c) Calibration curve for NSE determination using this aptamer based SPR assay. Error bars are standard deviation for three independent determinations.

Under the optimal experimental conditions, we achieved the sensitive analysis of NSE by using this aptamer coated sensor chip. SPR responses of aptamer coated chip to different concentrations of NSE ranging from 1.95 nM to 8 μM were measured (shown in Fig. 4). With the increase of NSE in solution, the relative SPR responses increased gradually. A good linear fitting was obtained between SPR relative responses and NSE concentrations in the range from 3.9 nM to 1 μM. The linear fitting regression equation was described as *y* = 0.0945 *x* + 2.4938 (*R*^2^ = 0.0987), where *y* was the SPR relative response unit, and *x* was the concentration of NSE. The detection limit of NSE was about 3.9 nM based on the signal-to-noise of 3 (S/N = 3). Though this SPR analysis, the dissociation constant (*K*_d_) was determined to be about 83 nM, which verifies the relatively high binding affinity of NSE to aptamer. The result is slightly different from that reported in the previous report (*K*_d_ ≈ 12 nM),^21^ which was measured by using the NSE-immobilized CM5 sensor chip. Through kinetic analysis, the binding rate constant and dissociation rate constant were determined to be 1.21 × 10^4^ Ms^−1^ and 1.004 × 10^−3^ s^−1^, respectively, which indicate the relatively fast association and slow dissociation of NSE with aptamer.

We also tested the stability of this aptamer-coated sensor chip. After storage for 3 months at 4 °C, NSE-induced SPR responses of the aptamer coated chip almost remained constant as the results tested on the first day (Fig. S2 in ESI†). The results indicate that the prepared aptamer coated chip can be used repeatedly and shows good stability.

Compared to some immunoassays,^9,37,38^ this proposed aptamer-based SPR assay introduced aptamer as affinity ligand, which shows advantages in ease of synthesis and labelling, and high stability over antibody. There are still limited aptamer based detection methods for NSE and ProGRP31-98. A sandwiched chemiluminescence aptasensor for NSE detection in serum samples was developed.^21^ By utilizing the signal amplification strategy of enzyme reaction, this aptasensor showed lower detection limit than our direct assay for NSE. Benefitting from the dual signal amplification effect of the synthesized coreshell nanoparticles, a sandwich structured electrochemical sensor showed extremely high sensitivity for NSE detection.^23^ As to ProGRP31-98, a label-free electro-chemiluminescence aptasensor allowed the detection of ProGRP31-98 as low as 17 nM, which is comparable to our proposed assay.^22^

In comparison with above aptamer based assays, the as-proposed assay showed limited sensitivity for NSE, as it adopted the direct format without using any signal amplification strategies. This direct assay overcomes the limitations of sandwich structured analysis and competitive assays. It offers notable benefits of simplicity, rapidity and low material consumption. Screening of aptamers with much higher affinity helps to improve the sensitivity further. Meanwhile, some nanoparticles, like gold nanoparticles (AuNPs) and magnetic nanoparticles, can be introduced in SPR sensor to increase response and improve sensitivity.^39,40^ This assay integrates the strengths of aptamer and SPR analysis, and is suitable for real time and high throughput analysis. The aptamer-coated sensor chip is reusable and presents high stability, showing great potential for applications.

### ProGRP31-98 detection

By using this aptamer-based SPR sensing strategy, we further achieved the detection of ProGRP31-98. Biotinylated aptamer against ProGRP31-98, named as ProGRP-48-5BioTEG, was immobilized on SA sensor chip. The immobilization amount of ProGRP-48-5BioTEG was about 303 RU (Fig. S3 in ESI†). After testing the influence of MgCl_2_ in running buffer on SPR responses toward ProGRP31-98, we found the PBS buffer containing 2 mM MgCl_2_ gave the highest relative SPR signal (Fig. S4 in ESI†). Therefore, 1 × PBS, pH 7.5, 2 mM MgCl_2_ and 0.1% Tween 20 was used as running buffer for ProGRP31-98 analysis.

**Fig. 5:**
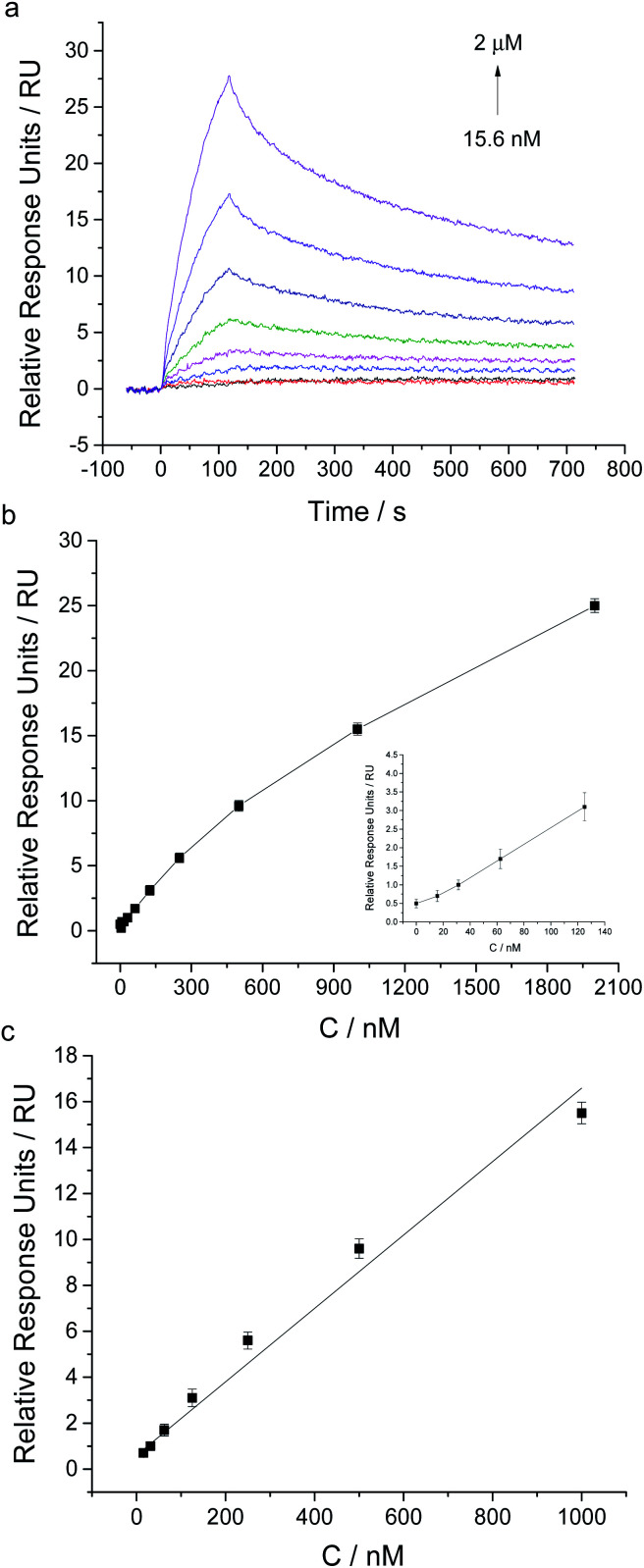
SPR analysis of different concentrations of ProGRP31-98 using the aptamer coated chip. (a) Sensorgrams of the injection of ProGRP31-98 at different concentrations ranging from 15.6 nM to 2 μM. (b) SPR response plot of the aptamer coated chip to different concentrations of ProGRP31-98 (inset: low concentrations ranging from 0 to 125 nM). (c) Calibration curve for ProGRP31-98 determination using this aptamer based SPR assay. Error bars are standard deviation for three independent determinations.

Different concentrations of ProGRP31-98 were injected for 120 s at a flow rate of 30 μL min^−1^, and the SPR responses were monitored in real time. As Fig. 5 showed, the SPR responses possessed a good linear relationship with the concentrations of ProGRP31-98 ranging from 15.6 nM to 1 μM (*y* = 0.0159 *x* + 0.6047 (*R*^2^ = 0.9811), where *y* was the SPR relative response unit, and *x* was the ProGRP31-98 concentration). The detection limit of ProGRP31-98 was about 15.6 nM (S/N = 3). The dissociation constant (*K*_d_) of ProGRP31-98 to aptamer was calculated to be 153 nM, and the binding rate constant and dissociation rate constant were determined to be 8.64 × 10^3^ Ms^−1^ and 1.321 × 10^−3^ s^−1^, respectively. The intermolecular interaction of ProGRP31-98 with aptamer also possesses a relatively fast association rate and a slow dissociation rate. The successful detection of ProGRP31-98 indicates that our direct aptamer based SPR assay allows the general detection of disease-related biomarkers.

### Specificity test

To evaluate the specificity of this proposed assay, we measured the SPR responses of anti-NSE aptamer coated chip toward some other biomarkers, including ProGRP31-98, alpha-fetoprotein (AFP), carcinoembryonic antigen (CEA), carbohydrate antigen 125 (CA125) and mucin-1 (MUC1). The injection of these biomarkers caused little increase of SPR signal, and the SPR signal induced by the mixture of these tested biomarkers with NSE was close to that of NSE sample (Fig. S5 in ESI†). The results present that this aptamer based SPR sensor possesses good specificity for NSE detection. The specificity of aptamer coated chip to ProGRP31-98 was also evaluated (Fig. S6 in ESI†). The results showed that these biomarkers caused weak SPR responses and the co-existence of these biomarkers and ProGRP31-98 did not interfere with ProGRP31-98 detection.

We also investigated the specificity of the sequence of aptamer by testing the DNA control. The control DNA was attached on SA sensor chip as the same procedure for aptamer immobilization. The immobilization amount of control DNA was about 337 RU (data was not shown). Then different concentrations of NSE were injected for 120 s at a flow rate of 30 μL min^−1^. As shown in Fig. S7 in ESI,† no significant change of SPR response was observed with the increase of NSE concentration. The result suggests that the control DNA cannot bind to NSE and the affinity of aptamer to NSE is sequence specific.

### Detection of NSE and ProGRP31-98 in diluted human serum

**Fig. 6:**
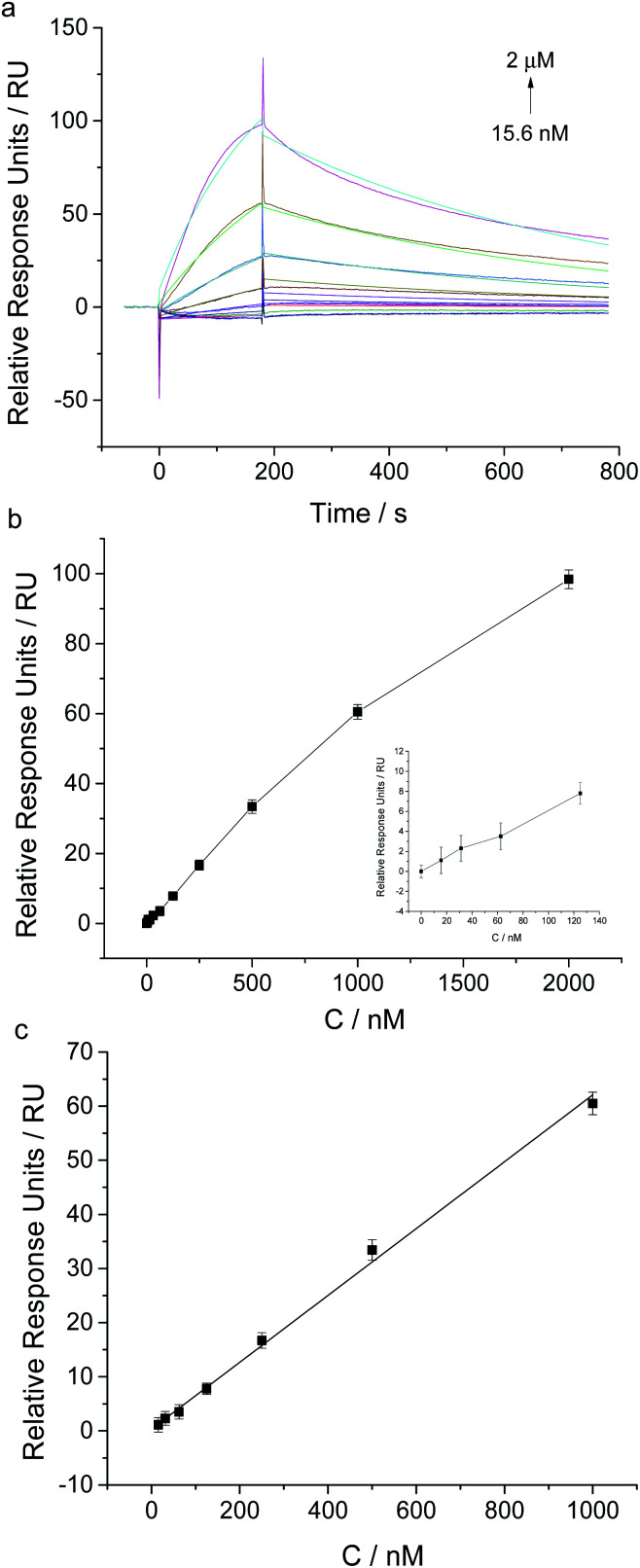
SPR analysis of different concentrations of NSE spiked in 100-fold diluted human serum. (a) Sensorgrams of the injection of NSE at different concentrations ranging from 15.6 nM to 2 μM. (b) SPR response plot of the aptamer coated chip to different concentrations of NSE (inset: low concentrations ranging from 0 to 125 nM). (c) Calibration curve for NSE determination using this aptamer based SPR assay. Error bars are standard deviation for three independent determinations.

In order to test the feasibility of this proposed assay in complex sample matrix, we spiked several concentrations of NSE in 100-fold diluted human serum and investigated the SPR responses of aptamer coated chip. As shown in Fig. 6, the relative response units and the concentrations of NSE ranging from 15.6 nM to 1 μM showed a good linear relationship (*y* = 0.0618 *x* + 0.3275, *R*^2^ = 0.9963, where *y* was the SPR relative response unit, and *x* was the concentration of NSE spiked in diluted human serum). The detection limit of NSE in 100-fold diluted human serum was determined to be 15.6 nM (S/N = 3), which was higher than that in running buffer. It may be caused by nonspecific binding of serum sample. As the real concentration of NSE in serum sample of a health person is below 0.3 nM, it is challenging for NSE direct detection in real serum samples by using our proposed assay. For further application in clinical test, some sample enrichment methods are required to meet the demand of NSE detection in serum samples. Therefore, combing with some sample pre-treatment methods, our aptamer based SPR assay is potential for NSE detection in human serum samples.

The feasibility of ProGRP31-98 analysis in 100-fold diluted human serum was also investigated. The injection of different concentrations of ProGRP31-98 caused relatively weak SPR responses (Fig. S8 in ESI†), indicating that a strong nonspecific binding of serum sample interfered with ProGRP31-98 detection. Further improve the affinity and specificity of aptamers contributes to improve the performance of NSE and ProGRP31-98 detection in complex sample matrix.

## Conclusions

In summary, we developed an aptamer based SPR assay in a direct assay format. The aptamer was loaded on SPR sensor chip and used as affinity ligand. With the flow of sample, SPR responses increased due to the affinity binding of target to aptamer coated chip. We measured the binding-induced SPR signals and achieved the sensitive detection of NSE and ProGRP31-98. The detection limit of NSE and ProGRP31-98 reached at 3.9 nM and 15.6 nM, respectively. Through SPR analysis, the affinity constants and kinetic parameters were also measured. This aptamer coated chip could be reused and stored for a long time at 4 °C. This developed aptamer based SPR assay is simple, rapid, and suitable for real time and high throughput analysis. Combing with some sample pre-treatment methods, this assay can be used for diluted human serum analysis. This assay shows potential in clinical disease-related biomarkers analyses.

## Author contributions

Linlin Sun proposed the idea of developing this aptamer-based SPR assay for NSE and ProGRP detection. Kemin Shen, Jianbin Zhang and Wenjuan Wan participated in the experiments with aptamer immobilization and quantitative analysis. Wenjun Cao and Zhijun Wang provided technical help with SPR experiments on Biacore instrument. Chongzheng Guo and Linlin Sun co-wrote the manuscript.

## Conflicts of interest

There are no conflicts to declare.

## Supplementary Material

RA-011-D1RA05041A-s001
