# Supplementary material for: Aptamer based surface plasma resonance assay for direct detection of neuron specific enolase and progastrin-releasing peptide (31-98)
Source: RSC Adv. 2021 Sep 29;11(51):32135–42. doi: 10.1039/d1ra05041a (PMC9041927; doi:10.1039/d1ra05041a)
Supplement: RA-011-D1RA05041A-s001 [file RA-011-D1RA05041A-s001.pdf]

Electronic Supplementary Material (ESI) for RSC Advances.
This journal is © The Royal Society of Chemistry 2021

Supplementary Materials

Aptamer based surface plasma resonance assay for direct detection of neuron specific enolase and progastrin-releasing peptide (31-98)

Linlin Sun,*[a] Kemin Shen,[a] Jianbin Zhang,[a] Wenjuan Wan,[a] Wenjun Cao,[a] Zhijun Wang,[b] Chongzheng Guo[a]

a. Department of Public Health and Preventive Medicine, Changzhi Medical College, Changzhi, Shanxi, 046000, China

b. Department of Chemistry, Changzhi University, Changzhi, Shanxi, 046011, China

* Corresponding author

E-mail: sunlinlin@czmc.edu.cn

Tel: +86-355-3151068

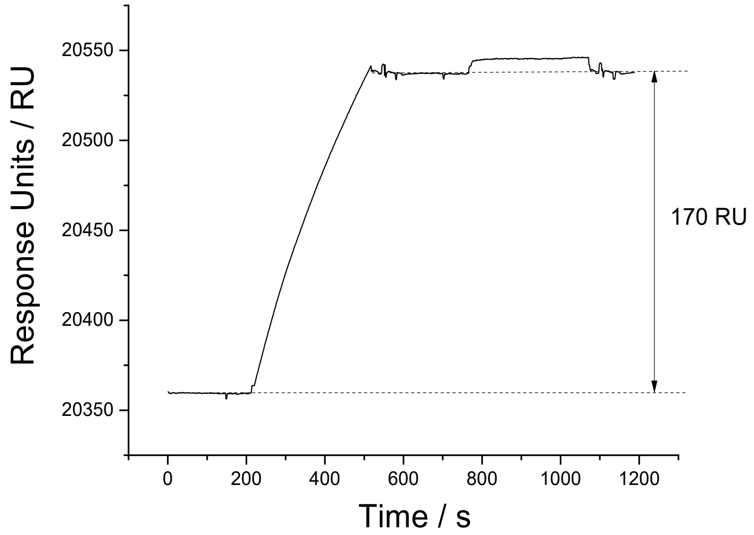

Fig.S1 Sensorgrams for the immobilization of biotinylated aptamer against NSE (NSE-Apt5-5BioTEG) on SA sensor chip

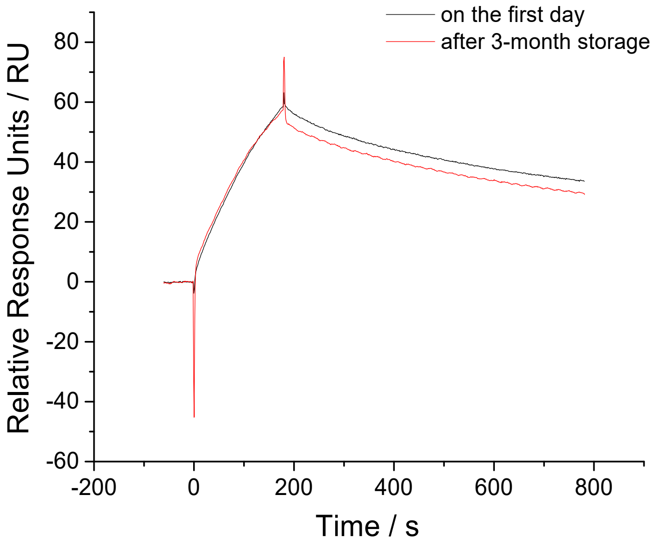

Fig.S2 SPR responses of the prepared aptamer coated chip on the first day and after 3-month storage at 4°C to NSE (500 nM) in running buffer

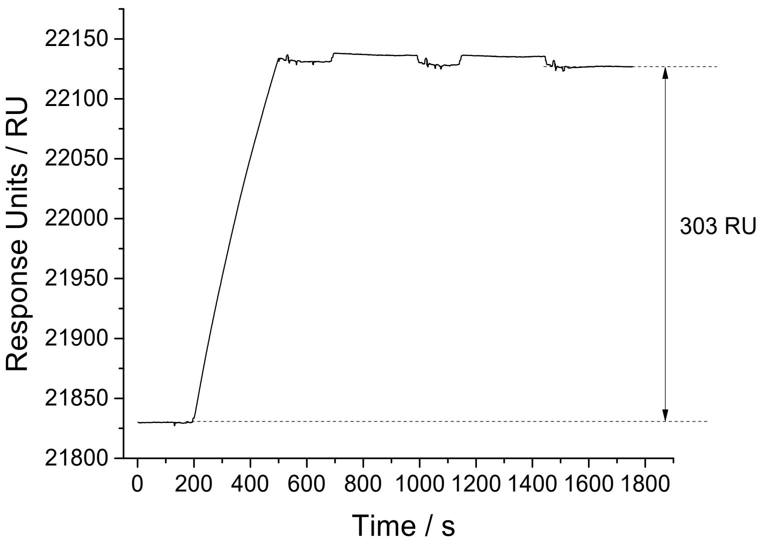

Fig.S3 Sensorgrams for the immobilization of biotinylated aptamer against ProGRP31-98 (ProGRP-48-5BioTEG) on SA sensor chip

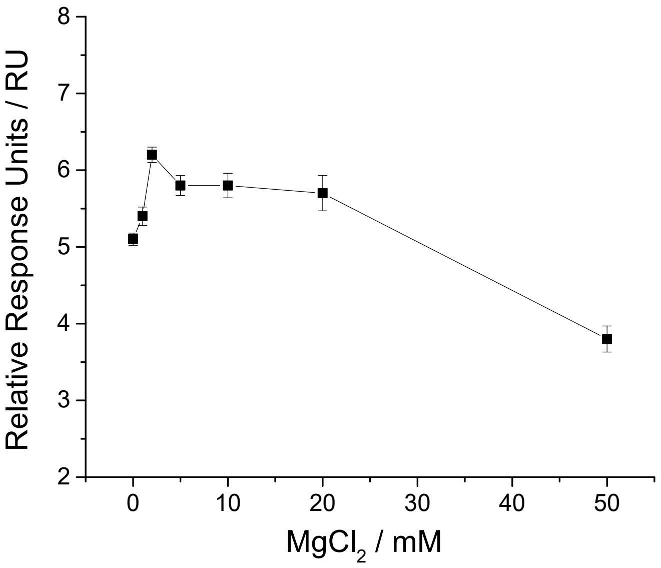

Fig.S4 Influence of $MgCl_2$ in running buffer on ProGRP31-98 detection, expressed as the relative SPR response units induced by ProGRP31-98 (500 nM) in running buffers containing 1×PBS, pH 7.5, 0.1% Tween 20 and different concentrations of $MgCl_2$. The relative response units were calculated by subtracting the response units of reference cell (flow cell 1) from that of sample cell (flow cell 2).

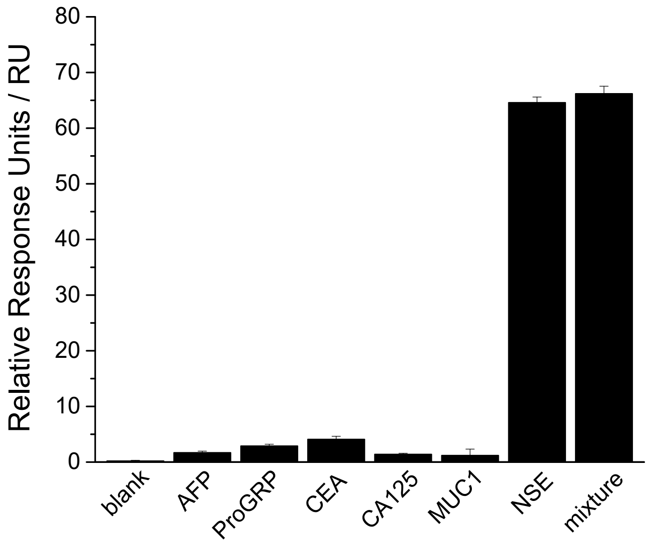

Fig.S5 Specificity test of the anti-NSE aptamer coated chip toward some other tumor markers, including AFP, ProGRP31-98, CEA, CA125, MUCI, NSE and the mixture of these biomarkers. Each of these biomarkers was tested at 500 nM.

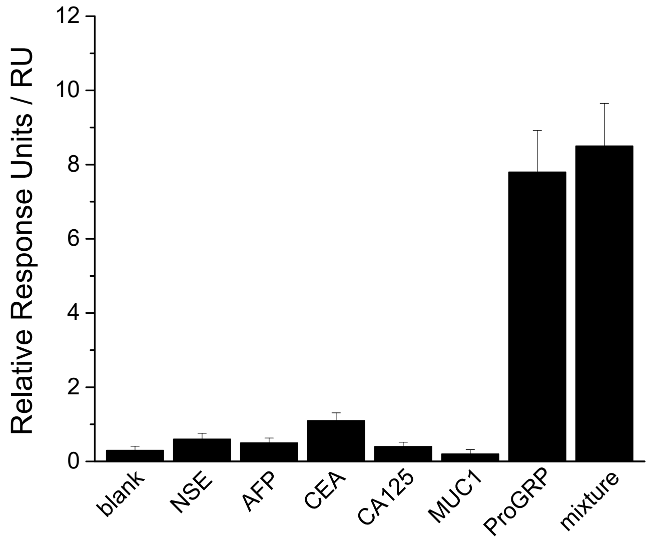

Fig.S6 Specificity test of the anti-ProGRP31-98 aptamer coated chip toward some other tumor markers, including NSE, AFP, CEA, CA125, MUC1, ProGRP31-98 and the mixture of these biomarkers. Each of these biomarkers was tested at 500 nM.

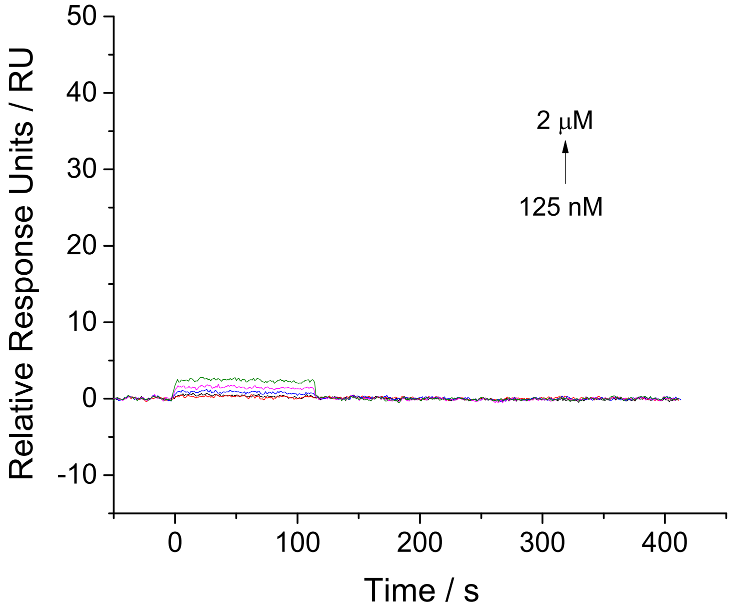

Fig.S7 Test of sequence specificity of the aptamer by using a control DNA coated chip, which was prepared as the same procedure for aptamer immobilization. Different concentrations of NSE ranging from 125 nM to 2 μM were injected for 120 s at a flow rate of 30 μL/min.

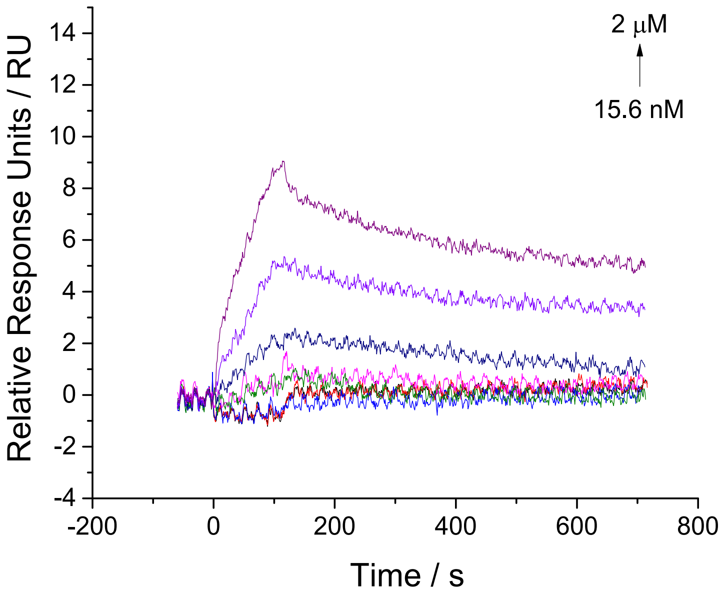

Fig.S8 SPR sensorgrams of different concentrations of ProGRP31-98 spiked in 100-fold diluted human serum.
